# Cultural Intelligence and Migration Intentions Among Nursing and Midwifery Students in Southeastern Region of Turkey: A Correlational Study

**DOI:** 10.1111/jan.16463

**Published:** 2024-09-20

**Authors:** Soner Berşe, Betül Tosun, Ezgi Dirgar, Ayla Yava

**Affiliations:** ^1^ Department of Nursing, Faculty of Health Sciences Gaziantep University Gaziantep Turkey; ^2^ Faculty of Nursing Hacettepe University Ankara Turkey; ^3^ Department of Midwifery, Faculty of Health Sciences Gaziantep University Gaziantep Turkey; ^4^ Department of Nursing, Faculty of Health Sciences Hasan Kalyoncu University Gaziantep Turkey

**Keywords:** brain drain, cultural intelligence, educational impact, intention to migrate, midwifery students, nursing students

## Abstract

**Aim:**

This study explores the relationship between cultural intelligence and attitudes towards the intention to migrate among nursing and midwifery students.

**Methods:**

Using a correlational design, data were collected from 804 students through the Student Descriptive Form, The Attitude Scale for Brain Drain in Nursing Students and the Cultural Intelligence Scale. The analysis was conducted using IBM SPSS Statistics and AMOS, employing structural equation modelling and analysis of variance.

**Results:**

The majority of participants were female (84.8%) and Turkish citizens (89.1%). The findings showed that both cultural intelligence levels and attitudes towards brain drain were above average. A positive correlation was identified between cultural intelligence and attitudes towards the intention to migrate.

**Conclusion:**

Enhancing cultural intelligence through intercultural education, overseas experiences and multilingual proficiency is essential. The study underscores the critical need for policy reforms aimed at improving working conditions in low‐income countries and addressing the challenges posed by the intention to migrate.

**Implications for the Profession and/or Patient Care:**

Incorporating cultural intelligence training into nursing and midwifery curricula is essential for preparing students to work in multicultural healthcare settings. Educational reforms are needed to equip future healthcare professionals with the skills required for diverse patient care. Additionally, policy interventions aimed at improving working conditions and offering better incentives in low‐income regions are critical to mitigating brain drain by addressing the root causes of migration among skilled healthcare workers.

**Impact:**

The study underscores the role of cultural intelligence in shaping the migration intentions of nursing and midwifery students. It highlights how higher levels of cultural intelligence correlate with more positive attitudes towards migration, suggesting that culturally adept healthcare professionals may be more inclined to seek international opportunities.

**Reporting Method:**

This study adhered to the STROBE guidelines for reporting observational studies.

**Patient or Public Contribution:**

No patient or public contribution.

## 1 Introduction

The wealth of many countries is measured not only by their natural resources, industrialisation and labour force potential but also by their commitment to education and science. Human capital is considered the most critical investment for national production and development (Elveren and Toksöz 2019; Güngör and Tansel 2014; Najib et al. 2019). The loss of this crucial investment, referred to as ‘brain drain’, represents the migration category with the highest increase in recent years in international migration movements (Oncu et al. 2018; Tosunoz and Nazik 2022).

This phenomenon has profound implications for both developed and developing countries, affecting healthcare systems, educational institutions and economic stability on a global scale. Understanding the factors that contribute to intention to migrate, particularly in the healthcare sector, is essential for formulating policies that can mitigate its adverse effects internationally. Brain drain involves the international transfer of resources whose capital lies in human intelligence. It often describes the migration of highly educated and skilled individuals from developing countries to developed countries (Munikar and Thapa 2019; Okafor and Chimereze 2020). The primary motivation behind this migration is the pursuit of better opportunities, including favourable working conditions, career advancement and lucrative salaries after specialisation (Kadel and Bhandari 2019). Developed countries prioritise investing a significant portion of their budgets in education and nurturing young talent, thereby becoming attractive destinations for skilled professionals. In this context, the intention to migrate has recently gained significant momentum as an international movement (Docquier 2014; Kizito et al. 2015; Oncu et al. 2018).

Cultural intelligence can be defined as an outsider's natural ability to comprehend, evaluate and interpret unfamiliar actions, behaviours, sounds, gestures, facial expressions, tones and accents the way that person's compatriots would (Majda et al. 2021; Rahimaghaee and Mozdbar 2017).

## 2 Background

Health professionals are among the most seriously affected occupational groups by the intention to migrate. Although the global migration of nurses and midwives is not a new phenomenon, it has intensified due to growing global demand for these professions (Oda, Tsujita, and Irudaya Rajan 2018; Tosunoz and Nazik 2022). Intention to migrate among health professionals has been steadily increasing since the mid‐1970s (Oda, Tsujita, and Irudaya Rajan 2018; Okolo and Ayah Iruo 2021). Future projections suggest that this trend will persist, driven by a widening gap between supply and demand (Aluttis, Bishaw, and Frank 2014; Mosuela and Mosuela 2020). In the 1970s, only 5% of nurses worked outside their country of origin, while recent years have seen a 60% increase in the number of migrant doctors and nurses, clearly illustrating the scale of the issue (Yeates and Pillinger 2018). In countries such as the United States, Canada, Germany and the United Kingdom, the proportion of immigrant healthcare personnel working in healthcare services has significantly increased (Yeates and Pillinger 2018). The loss of skilled nurses and midwives in countries experiencing brain drain negatively affects the quality and outcomes of care provided, increases the workload of healthcare workers and leads to shortages and inequalities in access to healthcare services (Hashish and Ashour 2020; Peters, Palomo, and Pittet 2020; Tosunoz and Nazik 2022). It is evident that assessing the attitudes of nursing and midwifery students towards the intention to migrate and associated factors and predictors is vital for healthcare system sustainability, quality, and productivity, since they represent a significant part of the healthcare workforce and aspire to become professional caregivers (Demiray, İlaslan, and Açıl 2020; Ndiangui 2021).

An employee with cultural intelligence will perceive individuals from different cultures as if they belonged to that culture, which will positively affect the quality of the service provided and foster better relationships with coworkers (Özkol Kılınç and Öztürk 2020). In this regard, it is of utmost importance for future nurses and midwives to develop cultural intelligence (Bertoli and Moraga 2015; Czaika and Parsons 2017; Dustmann and Görlach 2016). Investigating the relationship between cultural intelligence levels of healthcare workers who are increasingly inclined and taking action to migrate to work in developed countries could offer valuable insights for future planning. This study aimed to explore the relationship between cultural intelligence levels and attitudes towards the intention to migrate among nursing and midwifery students.

## 3 Methods

### 3.1 Sample and Setting

This study employed a descriptive and correlational design and was conducted from January to April 2023. It focused on undergraduate students pursuing degrees in nursing and midwifery. The research setting encompassed both a private and a public university located in Southeastern Anatolia, Turkey. This region is known for its cultural diversity and a significant immigrant population, primarily due to its proximity to the Syrian border. The study cohort included the entire body of undergraduate students registered in nursing and midwifery programs at the two universities for the academic year 2022/2023, totalling 1417 individuals. Sample size was calculated using the G*Power software package. As no study investigating cultural intelligence in relation to intention to migrate was identified in the literature, the sample size was determined to be at least 698, with a 95% confidence interval, *α* = 0.05 and (1 − *β*) = 0.80 power, assuming a Cohen's *d* (effect size) of 0.2 (Cohen 1988). All students were invited to participate in this study. Ultimately, the study was completed with 804 students (56.73% of the population), who agreed to participate and responded to the online questionnaire with no missing information.

### 3.2 Data Collection

After obtaining the required approvals, an electronic survey was distributed to each participant group via e‐mail. The data collection involved the use of Google Forms, a reliable online platform that allowed participants to conveniently submit their responses. To ensure the reliability of the data collected through the Google survey, several measures were implemented: a pilot test was conducted to identify potential issues, detailed instructions were provided, all questions were set as mandatory to prevent incomplete submissions, validation rules were included to ensure responses fell within expected ranges and continuous response monitoring was performed to identify any inconsistencies.

### 3.3 Data Collection Tools

Data were collected using the Student Descriptive Form, the Attitude Scale for Brain Drain in Nursing Students (ASBD) and Cultural Intelligence Scale (CQS).

### 3.4 Student Descriptive Form

This 14‐question form, developed by the researchers based on a literature review (Alan et al. 2022; Demiray, İlaslan, and Açıl 2020; Elveren and Toksöz 2019), collects descriptive information on sociodemographic and cultural characteristics.

### 3.5 The Attitude Scale for Brain Drain in Nursing Students (ASBD)

The 16‐item Attitudes Toward Migration Scale (ASBD) was developed by Oncu et al. (2018) to assess individuals' attitudes towards migration. The scale is structured into a single factor with two components: Push factors and pull factors. In the original study, the overall Cronbach's alpha was reported as 0.91, demonstrating strong internal consistency and reliability for the entire scale. The subscales also showed high reliability, with Cronbach's alpha values of 0.86 for the push factors and 0.88 for the pull factors. The ASBD utilises a 5‐point Likert scale ranging from 1 (strongly disagree) to 5 (strongly agree), with items 3 and 15 reverse coded. Scores on the scale can range from 16 to 80, with higher scores reflecting a more positive attitude towards migration and a greater inclination to migrate. In the current study, the overall Cronbach's alpha was found to be 0.93, with the push factors subscale showing a Cronbach's alpha of 0.89 and the pull factors subscale showing a Cronbach's alpha of 0.90.

### 3.6 Cultural Intelligence Scale

The Cultural Intelligence Scale (CQS), developed by Ang and van Dyne (2008), is a self‐report instrument designed to measure individuals' cultural intelligence. The scale comprises 20 items, utilising a 7‐point Likert‐type scale. It is divided into four subdimensions: the metacognitive subdimension, which includes four items assessing individuals' awareness and control over their cultural knowledge and cognitive processes; the cognitive subdimension, consisting of six items that measure knowledge of different cultures; the motivational subdimension, which comprises five items evaluating individuals' interest in interacting with people from different cultures and their self‐efficacy in this regard and the behavioural subdimension, which includes five items that assess the ability to exhibit appropriate verbal and nonverbal behaviours in cross‐cultural interactions. Scores on the CQS range from 20 to 140, with higher scores reflecting greater cultural intelligence.

In the original study by Ang and van Dyne (2008), the overall Cronbach's alpha for the CQS was 0.86, indicating satisfactory internal consistency. The Cronbach's alpha values for the subdimensions were reported as follows: 0.72 for the metacognitive subdimension, 0.86 for the cognitive subdimension, 0.76 for the motivational subdimension and 0.83 for the behavioural subdimension. In the Turkish validation study by Arastaman (2018), the overall Cronbach's alpha was reported as 0.92, demonstrating strong reliability.

In the present study, the overall Cronbach's alpha for the CQS was 0.929, indicating good internal consistency. The subdimensions yielded the following Cronbach's alpha values: 0.85 for the metacognitive subdimension, 0.89 for the cognitive subdimension, 0.87 for the motivational subdimension and 0.86 for the behavioural subdimension, all suggesting a high level of reliability and internal consistency.

### 3.7 Data Analysis

Data analysis was conducted using IBM SPSS Statistics for Windows, Version 23.0, and IBM SPSS AMOS Version 26.0. Descriptive statistics were presented as frequencies, percentages and mean ± standard deviation. Whether the data followed a normal distribution were checked using the Shapiro–Wilk test. Differences in mean scale scores across groups were examined using the Student's *t*‐test and one‐way analysis of variance as appropriate, with the Bonferroni correction applied to identify specific group differences. Furthermore, a path analysis was performed within the framework of structural equation modelling to explore relationships between the scales. This analytical approach allowed verification of the structural equation model and examination of the dataset's fit. The model fit to the collected data was evaluated using several fit indices, including Chi‐square/df (*χ* ^2^/df), root mean square error of approximation (RMSEA), goodness of fit index (GFI), normed fit index (NFI), nonnormed fit index (NNFI) and comparative fit index (CFI), providing a thorough assessment of the model's validity.

### 3.8 Ethical Considerations

Before initiating the study, ethical approval was obtained from the Institutional Review Board of Hasan Kalyoncu University, with which they are affiliated (Approval Date and Number: 01/01/2023‐2022/1). Additionally, institutional consents were obtained from the relevant bodies where the study was to be carried out, and permission to use the ASBD and CQS scales was secured from their respective proprietors through electronic communication. Participation in the survey was contingent upon students voluntarily providing informed consent, facilitated through an online mechanism. This study adhered strictly to ethical guidelines and principles outlined in the Declaration of Helsinki, ensuring compliance with international standards of research ethics.

## 4 Results

### 4.1 Descriptive Characteristics of the Students

The mean age of the study population was 20.52 ± 2.69 years. 84.8% of the students were female and 89.1% were Turkish citizens. Furthermore, 87.1% reported that Turkish was their native language, and 27% could speak another language fluently. Among the participants, 39.8% were first‐year students, and 75.6% were enrolled in a nursing program. Additionally, 10.2% of the students had previous experience travelling abroad, 71.4% intended to explore employment opportunities abroad after graduation and 38.1% reported actively following job postings from other countries. The students were asked to rate the perceived difficulty of working in a multicultural team and providing care for individuals from different cultures on a scale of 0–10, with mean scores presented in Table 1.

**TABLE 1:** Descriptive characteristics of the study sample (*n* = 804).

Demographic characteristics		
Gender		
Male	122	15.2
Female	682	84.8
Nationality		
Turkish	716	89.1
Foreign	88	10.9
Native language		
Turkish	700	87.1
Other	104	12.9
Fluently speaking another language		
Yes	217	27
No	587	73
Your school		
Public	604	75.1
Private	200	24.9
What grade are you in?		
1st year	320	39.8
2nd year	200	24.9
3rd year	162	20.1
4th year	122	15.2
Department		
Nursing	608	75.6
Midwifery	196	24.4
Have you been abroad before?		
Yes	82	10.2
No	722	89.8
Do you have plans to work abroad after graduation?		
Yes	574	71.4
No	230	28.6
Do you follow international job postings?		
Yes	306	38.1
No	498	61.9

	Mean ± SD	Min	Max
Age (years)	20.52 ± 2.69	18	52
Difficulty of working in a multicultural team	5.33 ± 1.99	1	10
Difficulty of providing care in a different culture	6.05 ± 2.21	1	10
ASBD total score	55.33 ± 11.44	16	80
ASBD push factors subdimension	15.17 ± 3.53	4	20
ASBD pull factors subdimension	40.15 ± 8.48	12	60
CQS total score	62.33 ± 12.01	20	100
Metacognitive	13.65 ± 2.87	4	20
Cognitive	16.46 ± 4.49	6	30
Motivation	16.10 ± 3.88	5	25
Behaviour	16.11 ± 3.65	5	25

### 4.2 Scale Scores and Group Comparisons Based on Students' Characteristics

The mean total scores were 55.33 ± 11.44 for the Attitude Scale for Brain Drain in Nursing Students (ASBD) and 62.33 ± 12.01 for the Cultural Intelligence Scale (CQS) respectively (Table 1).

Significant differences were observed between male and female students with respect to ASBD and CQS total scores. Male students showed higher mean scores for cultural intelligence and attitudes towards intention to migrate compared to female students (*p* = 0.006, *p* < 0.001 respectively) (Table 2).

**TABLE 2:** Comparison of CQS and ASBD scores based on the demographic characteristics of nursing and midwifery students.

Characteristics	ASBD push factors	ASBD pull factors	ASBD total	CQS metacognitive subscale	CQS cognitive subscale	CQS motivational subscale	CQS behavioural subscale	CQS total score
	Mean ± SD	Mean ± SD	Mean ± SD	Mean ± SD	Mean ± SD	Mean ± SD	Mean ± SD	Mean ± SD
Gender								
Female	3.78 ± 0.86	3.30 ± 0.69	3.42 ± 0.70	3.41 ± 0.71	2.68 ± 0.72	3.17 ± 0.76	3.20 ± 0.72	3.08 ± 0.58
Male	3.82 ± 0.98	3.55 ± 0.73	3.62 ± 0.76	3.43 ± 0.71	3.06 ± 0.76	3.48 ± 0.80	3.31 ± 0.75	3.30 ± 0.62
*t*/*p*	0.378/0.706	3.545/ 0.000*	2.738/0.006*	−0.387/0.699	5.192/0.000*	3.818/0.000*	−1.537/0.125	3.784/0.000*
Nationality								
Turkish	3.83 ± 0.87	3.22 ± 0.72	3.47 ± 0.71	3.42 ± 0.72	2.72 ± 0.75	3.21 ± 0.77	3.22 ± 0.72	3.11 ± 0.60
Other	3.43 ± 0.89	3.19 ± 0.77	3.29 ± 0.69	3.29 ± 3.69	2.88 ± 0.67	3.30 ± 0.77	3.19 ± 0.77	3.15 ± 0.58
*t*/*p*	4.054/0.000*	1.359/0.175	2.294/0.024*	1.599/0.110	1.900/0.058	1.127/0.262	0.332/0.741	0.586/0.558
Native language								
Turkish	3.83 ± 0.86	3.35 ± 0.70	3.47 ± 0.71	3.42 ± 0.72	2.71 ± 0.75	3.20 ± 0.77	3.22 ± 0.73	3.10 ± 0.60
Other	3.50 ± 0.93	3.27 ± 0.70	3.32 ± 0.73	3.33 ± 0.66	2.92 ± 0.69	3.33 ± 0.74	3.20 ± 0.69	3.17 ± 0.55
*t*/*p*	3.669/0.000*	1.184/0.237	2.005/0.045*	1.139/0.255	2.631/0.059	1.602/0.110	0.263/0.793	1.145/0.252
Do you speak another language fluently?								
Yes	3.77 ± 086	3.43 ± 0.69	3.51 ± 0.71	3.41 ± 0.72	2.94 ± 0.72	3.39 ± 0.74	3.24 ± 0.71	3.22 ± 0.60
No	3.80 ± 0.88	3.31 ± 0.70	3.43 ± 0.71	3.41 ± 0.71	2.66 ± 0.74	3.15 ± 0.77	3.21 ± 0.73	3.07 ± 0.59
*t*/*p*	0.412/0.680	2.087/0.037*	1.418/0.157	0.021/0.983	4.785/0.000*	3.929/0.000*	0.491/0.623	3.197/0.001
Department								
Nursing	3.79 ± 0.90	3.37 ± 0.71	3.48 ± 0.72	3.41 ± 0.72	2.81 ± 0.74	3.24 ± 0.76	3.25 ± 0.73	3.15 ± 0.60
Midwifery	3.78 ± 0.82	3.26 ± 0.68	3.39 ± 0.67	3.41 ± 0.70	2.53 ± 0.72	3.14 ± 0.80	3.12 ± 0.72	3.01 ± 0.56
*t*/*p*	0.117/0.860	1.970/0.049*	1.514/0.130	−0.072/0.943	4.629/0.000*	1.620/0.106	2.091/0.037*	2.863/0.004*
What grade are you in?								
1st year^a^	3.80 ± 0.81	3.30 ± 0.68	3.43 ± 0.67	3.41 ± 0.70	2.76 ± 0.72	3.26 ± 0.75	3.20 ± 0.71	3.12 ± 0.57
2nd year^b^	3.65 ± 0.96	3.30 ± 0.71	3.38 ± 0.74	3.29 ± 0.69	2.73 ± 0.71	3.14 ± 0.74	3.17 ± 0.68	3.05 ± 0.57
3rd year^c^	3.83 ± 0.90	3.44 ± 0.72	3.54 ± 0.74	3.59 ± 0.78	2.82 ± 0.84	3.29 ± 0.85	3.38 ± 0.80	3.23 ± 0.67
4th year^d^	3.94 ± 0.86	3.39 ± 0.71	3.53 ± 0.72	3.39 ± 0.66	2.58 ± 0.72	3.15 ± 0.77	3.13 ± 0.72	3.02 ± 0.57
*F*/*p*	3.109/0.026	1.974/0.116	2.078/0.102	5.108/0.002*	2.660/0.047*	1.742/0.157	3.549/0.014*	3.689/0.012*
	d‐b			c‐b	c‐d		c‐b.d	c‐a.b.d
Have you ever travelled abroad?								
Yes	3.64 ± 0.91	3.32 ± 0.70	3.40 ± 0.72	3.33 ± 0.75	2.90 ± 0.72	3.37 ± 0.77	3.21 ± 0.70	3.18 ± 0.61
No	3.81 ± 0.87	3.34 ± 0.70	3.46 ± 0.71	3.42 ± 0.71	2.72 ± 0.75	3.20 ± 0.77	3.22 ± 0.73	3.10 ± 0.59
	−1.568/0.117	−0.248/0.804	−0.667/0.505	−1.003/0.316	2.059/0.040*	1.937/0.053	−0.114/0.909	1.119/0.264
Do you have plans to work abroad after graduation?								
Yes	4.03 ± 0.72	3.60 ± 0.55	3.70 ± 0.56	3.45 ± 0.73	2.80 ± 0.73	3.35 ± 0.77	3.30 ± 0.72	3.19 ± 0.60
No	3.20 ± 0.95	2.71 ± 0.63	2.83 ± 0.67	3.31 ± 0.67	2.58 ± 0.75	2.89 ± 0.68	3.02 ± 0.71	3.91 ± 0.55
	11.769/0.000*	19.600/0.000*	17.399/0.000*	2.525/0.000*	3.867/0.000*	8.363/0.000*	4.888/0.000*	6.104/0.000
Do you follow international job postings?								
Yes	4.08 ± 0.75	3.74 ± 0.57	3.82 ± 0.58	3.55 ± 0.76	2.94 ± 0.76	3.48 ± 0.80	3.37 ± 0.73	3.31 ± 0.64
No	3.61 ± 0.90	3.10 ± 0.67	3.23 ± 0.69	3.32 ± 0.67	2.61 ± 0.71	3.05 ± 0.70	3.13 ± 0.71	2.99 ± 0.54
*t*/*p*	7.888/0.000*	14.439/0.000*	13.103/0.000*	4.357/0.000*	6.201/0.000*	7.716/0.000*	4.630/0.000*	7.148/0.000*
Have you taken a course on cultural care?								
Yes	3.76 ± 0.92	3.38 ± 0.73	3.48 ± 0.74	3.50 ± 0.79	2.95 ± 0.69	3.31 ± 0.81	3.38 ± 0.75	3.26 ± 0.63
No	3.80 ± 0.87	3.33 ± 0.70	3.45 ± 0.71	3.39 ± 0.70	2.70 ± 0.75	3.20 ± 0.76	3.19 ± 0.72	3.09 ± 0.59
*t*/*p*	−0.464/0.643	0.907/0.479	0.382/0.702	1.547/0.122	3.476/0.001*	1.494/0.136	2.562/0.008*	2.962/0.003*

*Note: t*: independent samples *t*‐test. *F*: one‐way ANOVA. c‐b: Bonferroni Correction: Applied for multiple comparisons; c‐b: Indicates a significant difference between 3rd year (c) and 2nd year (b) students; c‐d: Indicates a significant difference between 3rd year (c) and 4th year (d) students; c‐b.d: Shows that 3rd year (c) students differ significantly from both 2nd year (b) and 4th year (d) students; c‐a.b.d: Demonstrates that 3rd year (c) students are significantly different from 1st (a), 2nd (b), and 4th year (d) students. a, b, c, d correspond to the grade levels.

Abbreviations: ANOVA, one‐way analysis of variance; ASBD, Attitude Scale for Brain Drain; CQS, Cultural Intelligence Scale.

* *p* < 0.05.

Turkish citizens and native Turkish‐speaking students exhibited higher mean scores for the push factors subscale of ASBD (*p* < 0.001, *p* = 0.024 respectively) and ASBD total scores (*p* = 0.000, *p* = 0.045 respectively) than others. Moreover, the students who could speak another language fluently had higher mean scores for the ASBD pull factors, as well as the CQS cognition, CQS motivation subscales and CQS total scores (*p* = 0.037, *p* = 0.000, *p* = 0.000, *p* = 0.001 respectively) (Table 2).

Nursing students showed higher CQS total scores than midwifery students (*p* = 0.004). Significant differences were found between students who had taken courses on cultural care and those who had not in terms of CQS total scores, and CQS behaviour and cognition subscale scores (*p* = 0.001, *p* = 0.008, *p* = 0.003 respectively). Also, there was a significant difference in CQ total scores among students across different academic years (*p* = 0.012), with third‐year students displaying higher cultural intelligence levels than others, as shown by the Bonferroni correction (Table 2).

Significant differences were observed in ASBD and CQS total scores, as well as all subscale scores for the students who considered working abroad after graduation and those who followed international job postings than those who did not. The students with intentions to work abroad and those actively following job postings exhibited higher cultural intelligence levels and more positive attitudes towards intention to migrate than those who did not (*p* < 0.001 for all) (Table 2).

### 4.3 Results of Structural Equation Modelling

The relationship between ASBD and CQS scales was investigated using structural equation modelling. The model fit was assessed by comparing fit indices against ideal or acceptable values. The model's fit indices were above acceptable limits: GFI = 0.983, AGFI = 0.950, NFI = 0.979, RFI = 0.955, IFI (incremental fit index) = 0.983, TLI (Tucker–Lewis index) = 0.963 and CFI = 0.983. The parsimony‐adjusted measures, including PRATIO, PNFI and PCFI, were 0.467, 0.457 and 0.459 respectively. Error criteria, including RMSEA and FMIN values, were investigated, showing a RMSEA value of 0.079 and a FMIN value of 0.052 (Table 3).

**TABLE 3:** Fit indices for structural equation model analysis.

	*χ* ^2^/df	*p*	RMSEA	GFI	NFI	AGFI	CFI
Model	5.995	0.000	0.079	0.98	0.97	0.95	0.98

Abbreviations: AGFI, adjusted goodness of fit index; CFI, comparative fit index; GFI, goodness of fit index; NFI, normed fit index; RMSEA, root mean square error of approximation.

## 5 Discussion

### 5.1 Students' Intention to Migrate and Cultural Intelligence

In this study investigating the relationship between cultural intelligence and the intention to migrate among nursing and midwifery students, it was found that the students have a high tendency to migrate, along with positive attitudes towards migration and high cultural intelligence levels. In comparison to our findings, previous studies have reported lower or higher mean scale scores (Büyükbeşe and Yıldız 2016; Kant and Sevgi Ünal 2017; Yoğurtçu 2015), possibly related to the differences in the characteristics of the study populations and regional differences in educational settings. The notably high ASBD and CQS scores underscore the rapid communication and cultural interactions in today's globalised world.

### 5.2 Summary and Discussion of Key Findings

This study revealed that nursing students had a higher tendency to migrate and higher cultural intelligence levels than midwifery students. It has been reported that, compared to midwifery students, nursing students generally display more positive attitudes towards providing care to individuals from different cultures (Bal and Koç 2022; Öz et al. 2018). This may be explained by the fact that nursing students more frequently encounter patients from different cultures in clinical practice and receive more extensive education on cross‐cultural nursing care than midwifery students.

In this study, male students showed a higher tendency to migrate and higher cultural intelligence levels than female students. This finding is in line with previous reports (Darvish et al. 2013; Özkol Kılınç and Öztürk 2020; Tosunoz and Nazik 2022; Uludağ and Deveci 2018). This suggests that male students may have a better understanding of the cultural values across different cultures, including languages, religions, legal and economic conditions, nonverbal behaviours and arts, potentially contributing to their higher inclination towards migration. In addition, gender roles and associated obligations and responsibilities among female students may affect their decisions regarding migration (Tosunoz and Nazik 2022).

Several studies reported that aspirations to work abroad serve as a catalyst for increased intention to migrate (Asadi et al. 2017; Dziwornu, Yakar, and Temurçin 2016). The majority of participants in the present study expressed intentions to work abroad after graduation. It has been observed that students harbouring such aspirations and actively pursuing international job opportunities are more inclined to migrate and exhibit higher levels of cultural intelligence. Findings from other studies corroborate this trend (Poudel et al. 2018; Santric‐Milicevic et al. 2015; Seven and Adadıoğlu 2022). This concerning trend reflects the greater challenges faced by practising nurses and midwives in Turkey, a developing nation, compared to regions where migration is the preferred course of action.

This study found that speaking another language fluently positively affected the students' cultural intelligence levels and tendency to migrate. Language proficiency is an important factor when considering migration to another country, which can boost self‐confidence in diverse linguistic environments (Efendi et al. 2021; Lee 2016). A study involving nursing students has similarly reported a strong correlation between proficiency in a language other than their mother tongue and attitudes towards intention to migrate (Demiray, İlaslan, and Açıl 2020). The observation that fluency in other languages correlates with increased self‐confidence and tendency to migrate among students is noteworthy and emphasises its importance in career aspirations.

On the other hand, this study found that the students who had previously travelled abroad and those who received courses on cultural care scored higher in the cognitive subdimension of the CQS, with the latter also exhibiting higher total COS scores. Cultural encounters are meaningful experiences that facilitate the understanding of foreign cultures, enhancing knowledge, awareness and sensitivity towards cultural diversity (Sharifi, Adib‐Hajbaghery, and Najafi 2019). Previous overseas experiences may have positively influenced cognitive cultural intelligence of these students through exposure to different cultures (Demiray, İlaslan, and Açıl 2020). Additionally, literature findings support the notion that cultural intelligence levels can be developed and improved through education (Tosun et al. 2021).

### 5.3 Implications for Practice and Policy

The correlational analyses conducted in this study revealed a positive relationship between the students' cultural intelligence levels and their attitudes towards brain drain. Individuals with higher levels of cultural intelligence are more skilled in understanding and adapting to different cultures. Consequently, they are more inclined towards seeking job opportunities abroad and foreign educational prospects become more appealing to them. High cultural intelligence enables individuals to navigate and integrate into foreign cultures more effectively, thereby mitigating the challenges of being an expatriate (Poudel et al. 2018; Ramalu and Subramaniam 2019). This is a clear indication of how strong and accessible communication, interaction and mobilisation resources are in today's world. That being said, since developed countries attract healthcare professionals including midwives and nurses from developing countries by offering better living and working conditions, which results in an imbalanced distribution of healthcare workforce worldwide, there is a need to formulate proactive policies to reverse the current pattern of migration among healthcare workers.

### 5.4 Limitations

This study has several limitations that should be considered when interpreting the findings. While the relatively large sample size of 804 students was deemed sufficient for the study setting based on power analysis, the sample may not fully represent the broader population of nursing and midwifery students across the entire country. The strength of this study lies in its robust sample size and rigorous power analysis conducted to ensure statistical validity for the targeted region. However, further studies involving a more diverse and larger sample from various regions and countries are required to enhance the generalisability of our findings.

Additionally, this study could not objectively examine and account for potential confounding variables such as socio‐economic status and language proficiency levels. These factors may have a significant impact on both cultural intelligence and intention to migrate among nursing and midwifery students. Future studies should consider incorporating these variables to provide a more detailed understanding of the relationships under investigation.

## 6 Conclusions

Cultural intelligence plays a significant role in shaping the attitudes of nursing and midwifery students towards migration. Higher levels of cultural intelligence correlate with more positive attitudes towards migration, and culturally adept healthcare professionals are more inclined to seek international opportunities. To balance this and prevent brain drain, it is essential to implement educational programs that enhance cultural intelligence while simultaneously taking socio‐economic and political measures to address the issue.

Policies aimed at improving working and living conditions, particularly in low‐income regions, and providing better incentives for healthcare professionals are crucial. By doing so, the tendency of skilled healthcare workers to seek international opportunities can be reduced, encouraging them to stay and serve in their own countries. Incorporating cultural intelligence training into nursing and midwifery curricula can enhance students' preparedness for multicultural healthcare settings. This indicates a need for educational reforms to better equip future healthcare professionals with the skills needed to navigate diverse patient populations.

Additionally, promoting language proficiency programs will help students develop the confidence and skills needed to work in multicultural environments, thereby decreasing their tendency to migrate in search of better opportunities. Future research could explore the nuanced impacts of socio‐economic status and language proficiency on the intention to migrate and cultural intelligence among nursing and midwifery students.

## Author Contributions

B.T., S.B., E.D. and A.Y. contributed to the concept/design. S.B. and E.D. were involved in data collection. S.B., B.T. and E.D. performed data analysis and interpretation. B.T., E.D., S.B. and A.Y. contributed to manuscript drafting. B.T. and A.Y. contributed to the critical revision of the manuscript.

## Ethics Statement

To conduct the study, the researchers obtained an ‘Ethics Committee Approval’ from the noninterventional research ethics committee of the university they were affiliated with (Date: 01.01.2023 Decision Number: 2023/01), institutional permission from the institutions where the study would be conducted and permission to use the scale from the scale owner via email. Informed consent was obtained online from students who voluntarily participated in the study. This study was conducted in accordance with the ethical rules and principles of the International Helsinki Declaration.

## Conflicts of Interest

The authors declare no conflicts of interest.

### Peer Review

The peer review history for this article is available at https://www.webofscience.com/api/gateway/wos/peer‐review/10.1111/jan.16463.

## Supporting information


Data S1.

## Data Availability

The data associated with the study and analyses will be made available by the authors upon appropriate request for academic and scientific review purposes. The sharing of these data will be conducted in accordance with legal requirements and ethical principles pertaining to the protection of personal data.
